# Epidemiological overview – 18 years of ICU hospitalization due to trauma in Brazil

**DOI:** 10.11606/s1518-8787.2019053001178

**Published:** 2019-09-18

**Authors:** Maicon Henrique Lentsck, Ana Paula Sayuri Sato, Thais Aidar de Freitas Mathias

**Affiliations:** I Universidade Estadual de Maringá. Programa de Pós-Graduação em Enfermagem. Maringá, PR, Brasil; II Universidade de São Paulo. Faculdade de Saúde Pública. Departamento de Epidemiologia. São Paulo, SP, Brasil; III Universidade Estadual de Maringá. Departamento de Enfermagem. Maringá, PR, Brasil

**Keywords:** Wounds and Injuries, epidemiology, Intensive Care Units, trends, Critical Care, Time Series Studies

## Abstract

**OBJECTIVE:**

Assess the magnitude and trend of hospitalization rates due to traumatic injuries in intensive care units (ICU) in Brazil from 1998 to 2015.

**METHODS:**

This is an ecological time-series study that analyzed data from the Hospital Information System. A trend analysis of hospitalization rates was performed according to diagnosis, sex and age using generalized linear regression models and Prais-Winsten estimation.

**RESULTS:**

Rates were higher among male patients, but increased hospitalization due to trauma among female patients influenced the ratio between both sexes. Falls and transport accidents were the most frequent causes of trauma. The average annual growth was 3.6% in ICU trauma hospitalization rates in Brazil, the highest growth was reported in the North region (8%; 95%CI 6.4-9.6), among women (5.4%; 95%CI 4.5-6.3), and among people aged 60 years and older (5.5%; 95%CI, 4.7-6.3). The most frequent causes of trauma are falls (4.5%; 95%CI 3.5-5.5) and care complications (5.4%; 95%CI 4.5-6.3). On the other hand, the annual hospital mortality rate due to trauma in ICU is 1.7% lower, on average (95%CI 2.1-1.3).

**CONCLUSION:**

An increase in ICU hospitalization rate due to trauma in Brazil may be the result of some factors, such as an increasing number of accidents and cases of violence, the implementation of pre-hospital care, and improved access to care, with more beds in ICU. In addition, population aging is another factor, as a greater increase in hospitalization was observed among people aged 60 years and older.

## INTRODUCTION

Trauma from external causes remains the major cause of death and disability among people aged 5 to 29 years^1^ , although aging may help increase trauma hospitalization rates among older adults. In Spain, from 2000 to 2010, the annual increase in rates among older adults was 1.1% for male and 0.9% for female patients^2^ ; in the United States, hospitalization due to trauma accounted for 4.4% of the total rate from 2000 to 2011, declining for children and young people and stable for older adults^3^ . In 2015, trauma accounted for 10.1% of the global burden of disease^4^ . Depending on the severity, it causes sequelae or even death.

In 2017, the Global Burden of Disease (GBD) study estimated that nonfatal trauma from falls and traffic accidents generated short- and long-term disability in 226.2 million people^5^ . And fatal injuries represented 8% of mortality in the world, affecting 4.48 million people, a 2.3% increase when compared to 2007 and a global mortality rate of 57.9 for every 100,000 inhabitants^6^ . Also in 2007, trauma accounted for 11.9% of 1.65 billion potential years of life lost (PYLL)^6^ .

In addition to increased rate, the severity of traumatic injuries is also increasing. In Canada, from 2002 to 2009, growth of hospitalization rate due to severe trauma in people aged 65 years and older was 22%, and 10% among young people^7^ . This trend of increased severity of different types of trauma was also reported in Spain^2^ . Increased severity of trauma involves improvements in health services and specialization of the multiprofessional team and requires hospitalization in intensive care units (ICU). To learn more about morbidity due to trauma in Brazil, the Hospital Information System (SIH) of the Brazilian Unified Health System (SUS) was analyzed, which is the database about hospitalization in the country. This analysis showed that hospitalization due to external causes increased 37.3% between 2002 and 2011, and the most frequent causes were falls (41%) and traffic accidents (15%)^8^ .

A significant number of trauma victims needs intensive care that is essential for their survival^9^ . In the United States, in 2013, 33.8% of trauma patients were in ICU, with a higher incidence among people aged 80 years and older (7.8 per 1,000 inhabitants)^10^ . Studies on ICU hospitalization due to trauma are critical for trauma care planning and organization and for the development of prevention programs^11^ .

With an increase in traffic accidents and violence, particularly involving aggression^12^ in large urban areas, ICU hospitalization due to trauma may also increase. Intensive care, especially in hospitalization, proportionally represents the highest cost of care network for the health sector and society. Considering the burden of traumatic injuries on morbidity and mortality in the population and that a significant number of victims is hospitalized, the ICU hospitalization rates in Brazil should be studied, identifying trends of this event in regions and states, and the profile by sex, age, type of external cause leading to hospitalization, length of stay, and hospital mortality. These studies may support planning and monitoring of public policies. Therefore, this study aimed to assess the magnitude and trend of ICU hospitalization rates due to traumatic injuries in Brazil from 1998 to 2015.

## METHODS

This is an ecological time-series study that analyzed data from ICU admissions due to trauma funded by SUS, based on hospital admission authorizations in the SIH-SUS from January 1998 to December 2015 in Brazil. The start of time series was selected according to the International Classification of Diseases, 10th edition (ICD-10), for hospital morbidity in Brazil, based on the publication of the decree that requires the injury code entry in the “primary diagnosis” field and the external cause in the “secondary diagnosis” field in the hospital admission authorizations^13^ . The end of time series refers to the latest hospitalization recorded in the data collection period.

Through the electronic address of the Department of Data Processing of SUS (DATASUS), monthly files were generated for each state and year of the time series. Records indicating ICU admission and primary diagnosis of injuries and poisoning (Chapter XIX of ICD-10) were selected for the groups: injury of head (S00.0-S00.9); injury of neck (S10.0–S10.9); injury of thorax (S20.0–S20.8); injury of abdomen, lower back, and pelvis (S30.0-S30.9); injury of upper limbs (S40.0-S60.9); injury of lower limbs (S70.0–S90.9); multiple body regions (T00.0–T07.0); unspecified body regions (T08.0–T14.0); foreign body effect through natural orifice (T15.0–T19.0); burns and corrosion (T20.0–T32.0); effects of cold (T33.0–T35.0); intoxication due to use of drugs, medications and biological substances (T36.0–T50.0); toxic effects of non-medicinal substances (T51.0–T65.0); other effects of external causes (T66.0–T78.0); some early complications of trauma (T79.0); complications of medical and surgical care (T80.0–T88.0), and sequelae of trauma (T90.0–T98.0). This selection resulted in a database with 790,884 admissions. Population data were collected via electronic address of the Brazilian Institute of Geography and Statistics (IBGE), based on population censuses, population counts and intercensal estimates.

Admissions due to traumatic injuries were analyzed by means of absolute and relative numbers, and relative difference. Rates per 100,000 inhabitants were calculated, categorized by sex, age and external cause, according to regions and states, standardized by the direct method, and using population of the 2010 census. Admissions due to external causes correspond to the secondary diagnosis and are codified according to Chapter XX of ICD-10 (V01-Y98); 7,542 admissions (0.9%) were lost due to lack of records. The means of length of stay and hospital mortality rates were calculated for every 100 admissions due to traumatic injuries.

A trend analysis was performed using generalized linear regression, considering hospitalization rates as a dependent variable (Y) and calendar years as an independent variable (X). The Prais-Winsten procedure was used to correct the effect of the first-order temporal autocorrelation of residues, which, according to the Durbin-Watson statistic, evaluates the presence of autocorrelation. The test is scored 0 to 4, where 2 means absence of serial autocorrelation^14^ .

Time series smoothing used the third-order moving average model. Then, log transformation was performed, and dispersion and autocorrelation diagrams were built. The Prais-Winsten regression model was used to identify whether the behavior of the rates was stable (p > 0.05), decreasing (p < 0.05 and negative β_1_ regression coefficient), or increasing (p < 0.05 and positive β_1_ regression coefficient). After modeling, the average annual variation of hospitalization rates was calculated for the regression coefficient using the equation: (-1 + 10^^^b)x100, and the respective 95% confidence intervals (95%CI) using the formula b±tSE, where t is the tabulated value for the t test and SE is the value of standard error of the regression coefficient^15^ . The tables presented data of 1998, 2007, and 2015, and the relative difference was calculated between the start and end years. For the trend analysis, Stata version 13 was used. The research ethics committee qualified this study for exemption of analysis according to Decree no. 466 of 2012 from the Brazilian National Health Council, considering the study used secondary data of public access.

## RESULTS

From 1998 to 2015, while all-cause hospitalization rates funded by SUS decreased 28.5%, ICU admissions increased 21.4% ( Table 1 ). When ICU hospitalization rates are analyzed by primary diagnosis, a higher growth was observed for conditions originated in the perinatal period (173.8% growth), followed by hospitalization due to trauma (91.8% increase) ( Table 2 ). Hospitalization due to trauma increased mainly in the North and Northeast regions – 296.9% and 167.1%, respectively. On the other hand, hospital mortality decreased 25.1% – in the North region a 33.5% reduction was reported ( Table 1 ). Head, hip and thigh traumas were the most frequent, with rates of 9.7 and 6.9 for every 100,000 inhabitants, respectively. The highest growth in the study period were in burns and corrosions (4,566.7%) and complications of medical and surgical care (466.7%) ( Table 2 ).

**Table 1:** Hospitalization rates for all causes and all types, for all causes in ICU and due to trauma in ICU, and mortality rates due to trauma in ICU, by regions in Brazil and year. Brazil, 1998, 2007 and 2015.

Admissions	1998		2007		2015		Relative diff.^b^
		
	n	Rate^a^	n	Rate^a^	n	Rate^a^	
All causes, all types							

Brazil	12,248,631	7,717.2	11,733,572	6,052.6	11,623,829	5,515.1	-28.5
North	851,777	7,176.6	996,349	6,494.0	951,726	5,447.0	-24.1
Northeast	3,601,756	7,962.2	3,277,182	6,278.9	3,120,990	5,518.0	-30.7
Southeast	4,904,176	7,111.5	4,671,206	5,792.6	4,660,086	5,434.8	-23.6
South	2,020,358	8,364.4	1,873,754	6,778.8	2,003,708	6,854.9	-18.0
Midwest	870,564	7,917.9	915,081	6,770.3	887,319	5,746.1	-27.4

All causes in ICU							

Brazil	381,642	275.7	511,989	278.1	705,181	334.8	21.4
North	5,911	73.5	19,796	158.4	29,996	215.4	193.1
Northeast	65,740	165.4	91,134	178.3	138,643	248.7	50.4
Southeast	178,310	293.7	246,763	302.7	334,322	336.2	14.5
South	98,640	456.8	119,607	431.0	155,431	484.9	6.2
Midwest	33,041	392.8	34,689	286.2	46,789	317.4	-19.2

Trauma^c^ in ICU							

Brazil	24,883	17.1	42,834	23.4	67,705	32.8	91.8
North	606	6.5	1,427	10.9	3,810	25.8	296.9
Northeast	3,003	7.3	6,280	12.7	10,763	19.5	167.1
Southeast	13,175	21.0	23,029	28.6	33,268	36.0	71.4
South	5,905	26.4	9,725	35.4	15,567	50.4	90.9
Midwest	2,144	22.8	2,373	18.7	4,297	29.0	27.2
Hospitality mortality	n	Rate^d^	n	Rate^d^	n	Rate^d^	Relative diff.^b^

Trauma in ICU							

Brazil	5,349	21.5	8,626	20.1	10,920	16.1	-25.1
North	170	28.1	294	20.6	711	18.7	-33.5
Northeast	781	26.0	1,598	25.4	2,011	18.7	-28.1
Southeast	2,904	22.0	4,407	19.1	5,079	15.3	-30.5
South	1,117	18.9	1,750	18.0	2,302	14.8	-21.7
Midwest	377	17.6	577	24.3	817	19.0	8.0

^a^ Rates standardized according to the Brazilian population of the 2010 census, per 100,000 inhabitants/year.

^b^ Relative difference between the rates of start/end years (1998 and 2015).

^c^ Primary diagnosis of hospitalization in Chapter XIX (injury, poisonings and some other consequences of external causes, codes S00-T98) of the International Classification of Diseases, 10th edition (ICD-10).

^d^ Hospital mortality rate: ratio between the number of deaths due to trauma in ICU and ICU hospitalization due to trauma, multiplied by 100.

**Table 2:** Hospitalization rate for all causes in ICU according to primary diagnosis of hospitalization and year. Brazil, 1998, 2007 and 2015.

Primary diagnosis^a^	1998		2007		2015		Relative diff.^c^
		
	n	Rate^b^	n	Rate^b^	n	Rate^b^	
Circulatory system diseases	129,248	105.7	156,919	90.8	193,810	89.0	-15.8
Respiratory system diseases	51,917	37.0	67,013	36.2	83,986	39.6	7.0
Perinatal conditions	31,269	14.1	66,631	29.8	75,492	38.6	173.8
Injury and poisoning	24,833	17.1	42,834	23.4	67,705	32.8	91.8
Head	10,822	7.1	16,497	8.9	20,145	9.7	36.6
Hip and thigh	3,204	2.8	6,613	3.8	14,725	6.9	146.4
Abdomen	2,057	1.4	3,995	2.1	5,468	2.7	92.9
Multiple regions of the body	2,244	1.5	646	0.3	1,904	0.9	-40.0
Non-medicinal poisonings	1,285	0.8	1,535	0.8	1,370	0.7	-12.5
Intoxication due to drugs	1,165	0.7	1,599	0.9	1,248	0.6	-14.3
Care complications^d^	830	0.6	3,275	1.8	7,267	3.4	466.7
Thorax	910	0.6	1,825	1.0	4,582	2.2	266.7
Burn and corrosion	73	0.03	1,950	1.0	2,925	1.4	4,566.7
Neck	441	0.3	1,144	0.6	1,393	0.7	133.2
Other parts^e^	1,802	1.2	3,755	2.1	6,678	3.2	166.7
Neoplasms	19,768	15.2	40,741	23.0	59,893	27.8	82.9
Other chapters of ICD-10	124,607	86.6	137,851	74.8	224,295	106.5	23.0

Total	381,642	275.7	511,989	278.1	705,181	334.8	21.4

ICD-10: International Classification of Diseases, 10th edition.

^a^ According to the chapters of ICD-10 and groups of Chapter XIX about injury and poisoning.

^b^ Rates standardized according to the Brazilian population of the 2010 census, per 100,000 inhabitants/year.

^c^ Relative difference between the rates of start/end years (1998 and 2015).

^d^ Complications of medical and surgical care not classified in other sections.

^e^ Trauma of shoulder and arm; elbow and shoulder; wrist and hand; knee and leg; ankle and foot; unspecified location; by foreign body penetration in natural holes; burn and corrosion; frostbite; other effects of unspecified causes; some early complications of trauma and sequelae of trauma, intoxication and other consequences.

The ICU hospitalization rates due to trauma in Brazil almost doubled, from 17.1 in 1998 to 32.8 for every 100,000 inhabitants in 2015, an average increase of 3.6% a year (95%CI 2.8–4,3) ( Figure ). Even with lower hospitalization rates, the highest growth was in the North region (8.0% a year, 95%CI 6.4–9.6) and in the States of Roraima (21.7% a year, 95%CI 7.4–37.8), Bahia (12.7% a year, 95%CI 9.3–16.3) and Rondônia (11.9% a year, 95%CI 0.0–25.3). Only the State of Paraíba reported reduction of 2.8% a year (95%CI 4.2–1.3). The Midwest region presented no trend for the rates (95%CI -1.3–3.3) due to oscillation of high rates in the State of Goiás. The South and Southeast regions reported in 2015 the highest hospitalization rates in the country: 50.4 and 36.0 for every 100,000 inhabitants, respectively. Even with an average annual increase of 3.3%, lower than those observed in the North and Northeast regions (8% and 5.3%, respectively), the South and Southeast regions presented high rates in the States of Paraná, Santa Catarina, São Paulo, and Minas Gerais (69.1, 45.6, 42.3, and 39.8, respectively) in 2015 ( Table 3 , Figure ).

**Figure:**
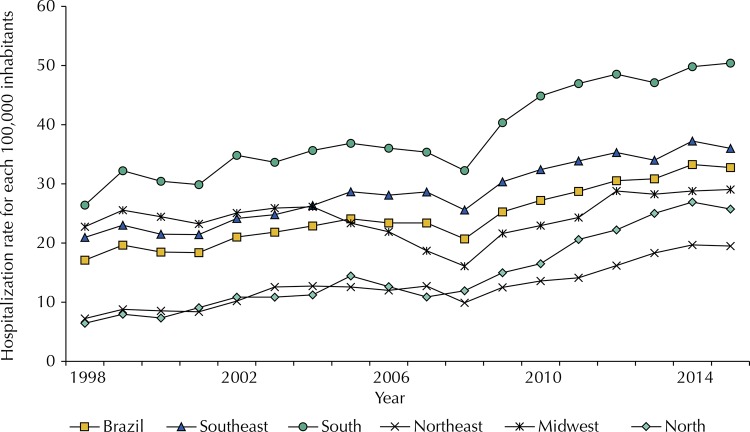
ICU hospitalization rates for each 100,000 inhabitants due to trauma. Brazil and regions, 1998–2015.

**Table 3:** ICU hospitalization rates due to trauma, average annual variation, confidence interval (95%CI) and trend. Brazil, regions and states, 1998 to 2015.

Brazil, regions and states	Rate^a^			Annual variation^b^	95%CI	Trend

	1998	2007	2015			
Brazil	17.1	23.4	32.8	3.6	2.8 – 4.3	Increasing
North	6.5	10.9	25.8	8.0	6.4 – 9.6	Increasing
Acre	8.5	22.5	37.7	3.6	1.7 – 5.6	Increasing
Amapá	6.5	11.8	13.2	6.2	-2.9 – 16.2	-
Amazonas	2.0	8.9	12.5	11.4	5.0 – 18.1	Increasing
Pará	5.3	8.8	25.5	11.0	8.6 – 13.3	Increasing
Rondônia	6.5	2.4	37.9	11.9	0.0 – 25.3	Increasing
Roraima	0.0	20.6	33.4	21.7	7.4 – 37.8	Increasing
Tocantins	14.3	15.9	34.4	5.4	0.2 – 10.8	Increasing
Northeast	7.3	12.7	19.5	5.3	3.7 – 6.8	Increasing
Alagoas	7.4	16.7	17.0	1.7	-2.0 – 5.4	-
Bahia	2.1	10.6	21.0	12.7	9.3 – 16.3	Increasing
Ceará	12.7	16.5	14.4	0.5	-2.7 – 3.8	-
Maranhão	3.0	7.6	17.3	9.5	4.6 – 14.7	Increasing
Paraíba	31.4	28.4	22.0	-2.8	-4.2 – -1.3	Decreasing
Pernambuco	4.0	5.9	25.2	10.4	3.9 – 17.2	Increasing
Piauí	5.7	9.4	18.0	7.0	3.5 – 10.6	Increasing
Rio Grande do Norte	4.2	28.3	14.1	7.9	0.6 – 15.8	Increasing
Sergipe	9.0	10.4	22.5	5.4	-0.0 – 11.1	-
Southeast	21.0	28.6	36.0	3.3	2.8 – 3.8	Increasing
Espírito Santo	13.3	22.9	33.0	5.3	2.9 – 7.9	Increasing
Minas Gerais	19.2	29.0	42.3	4.9	3.7 – 6.0	Increasing
Rio de Janeiro	12.9	9.6	19.1	2.2	-1.4 – 6.0	-
São Paulo	25.8	36.5	39.8	2.5	1.7 – 3.2	Increasing
South	26.4	35.4	50.4	3.3	2.5 – 4.1	Increasing
Paraná	39.4	44.5	69.1	3.5	1.8 – 5.3	Increasing
Rio Grande do Sul	16.8	26.6	38.4	3.0	1.3 – 4.7	Increasing
Santa Catarina	23.2	36.8	45.6	3.6	2.4 – 4.8	Increasing
Midwest	22.8	18.7	29.0	1.0	-1.3 – 3.3	-
Federal District	7.6	11.6	16.4	5.3	2.9 – 7.9	Increasing
Goiás	36.2	21.0	34.0	-1.2	-4.5 – 2.2	-
Mato Grosso	9.1	18.5	31.0	6.4	4.7 – 8.2	Increasing
Mato Grosso do Sul	18.9	20.5	28.2	2.6	0.5 – 4.8	Increasing

^a^ Rates standardized according to the Brazilian population of the 2010 census, per 100,000 inhabitants/year.

^b^ Average annual variation of hospitalization rates calculated from β_1_ of Prais-Winsten generalized linear regression model^11^.

ICU hospitalization rates due to trauma are twice as high for male patients, but the increase was higher among female patients (on average, 5.4% a year, 95%CI 4.5–6.3) than male patients (3.8% a year, 95%CI 3.1–4.5), which influenced the reduced ratio in the study period. The average annual growth rate was higher for people aged 60 years and older (5.5%, 95%CI, 4.7–6.3) ( Table 4 ).

**Table 4:** ICU hospitalization rates due to trauma by sex, age, external cause of injury, mean length of stay and hospital mortality, average annual variation, confidence interval (95%CI) and trend. Brazil, 1998–2015.

Characteristics	Rate^a^			Annual variation^b^	95%CI	Trend

	1998	2007	2015			
Sex						
Male	22.2	32.2	44.3	3.8	3.1 – 4.5	Increasing
Female	8.7	13.4	22.2	5.4	4.5 – 6.3	Increasing
Ratio M/F	2.6	2.3	2.0	-1.5	-2.1 – -1.0	Decreasing
Age (years)						
Up to 14	6.4	9.0	10.6	2.5	1.7 – 3.3	Increasing
15–29	17.4	23.3	29.7	3.2	2.5 – 3.8	Increasing
30–59	16.6	22.5	28.3	2.8	2.1 – 3.5	Increasing
60 and older	39.3	58.8	101.4	5.5	4.7 – 6.3	Increasing
External cause of injury^c^						
Care complications (Y40–Y84)	0.9	1.4	1.9	5.1	3.9 – 6.4	Increasing
Other accidental traumas (W20–X59)	3.0	4.2	7.0	4.6	3.4 – 5.8	Increasing
Falls (W00–W19)	5.0	7.6	10.8	4.5	3.5 – 5.5	Increasing
Sequelae of external causes (Y85–Y89)	0.6	0.7	1.2	4.5	1.8 – 7.3	Increasing
Other groups (Y35–Y36; Y90–Y98)	0.1	0.1	0.2	4.4	2.0 – 6.8	Increasing
Events of unspecified intention (Y10–Y34)	1.0	1.2	2.5	4.2	1.5 – 7.0	Increasing
Intentional self-inflicted injury (X60–X84)	0.2	0.5	0.5	4.0	1.5 – 6.7	Increasing
Transport accidents (V01–V99)	3.4	5.4	6.8	3.7	2.9 – 4.4	Increasing
Pedestrian (V01–V09)	0.8	1.5	1.3	4.0	2.3 – 5.9	Increasing
Cyclist (V10–V19)	2.0	3.1	3.5	3.2	2.8 – 3.6	Increasing
Motorcyclist (V20–V29)	1.2	1.8	2.8	4.3	3.1 – 5.5	Increasing
Vehicle occupant (V30–V79)	0.6	0.8	1.0	3.3	1.5 – 5.1	Increasing
Other (V80–V99)	0.6	1.0	1.4	3.1	0.2 – 6.1	Increasing
Aggression (X85–Y09)	1.1	1.4	2.2	3.2	1.5 – 4.9	Increasing
Mean of length of stay in ICU (days)	4.9	6.1	6.3	1.5	1.0 – 2.1	Increasing
Hospital mortality rate in ICU	21.5	20.1	16.1	-1.7	-2.1 – -1.3	Decreasing

Ratio M/F: ratio between male and female population.

^a^ Rates standardized according to the Brazilian population of the 2010 census, per 100,000 inhabitants/year.

^b^ Average annual variation of hospitalization rates calculated from β_1_ of Prais-Winsten generalized linear regression model ^11^.

^c^ Secondary diagnosis of hospitalization, Chapter XX, International Classification of Diseases, 10th edition (ICD-10).

Falls, traffic accidents and other accidents were the most frequent external causes of trauma for ICU admissions in 2015, with rates of 10.8; 6.8; and 7.0 for every 100,000 inhabitants, respectively. The hospitalization rates of motorcyclists and pedestrians had the greatest growth (4.3% and 4%, respectively). All external causes that led to ICU hospitalization showed growth in the study period, such as care complications (5.1%), other accidental trauma (4.6%), and falls (4.5%). The average ICU length of stay increased (1.5% a year, 95%CI 1.0–2.1); however, hospital mortality decreased, on average, 1.7% a year (95%CI 2.1–1.3) ( Table 4 ).

## DISCUSSION

An epidemiological analysis of SUS-funded hospitalizations in Brazil showed that all-cause hospitalizations declined in the study period, but the ICU admissions had an increase. From 1998 to 2015, trauma was the fourth cause of ICU admissions. Except for perinatal conditions, the relative increase in ICU hospitalization rates due to trauma was the highest, reported in all regions of the country, with annual growth averages higher in the North region and smaller in the Midwest region. Another change was observed in relation to sex and age, since the average increase in rates was higher among female patients and older adults.

Despite the heterogeneous characteristics of population morbidity and mortality, variation in ICU structure and capacity, and health care organization^10^ , ICU hospitalization due to trauma in the Brazilian public health system are similar to international data^16^ . A Swiss study on ICU hospitalization in 2008–2012 identified that traumas accounted for 44.3% of the total; the most prevalent were brain injury (64.4%) and thoracic trauma (59.8%), and the main causes were traffic accidents (40.4%) and falls (34.4%)^16^ .

The increase in external causes of ICU hospitalization due to trauma is similar to the hospital morbidity due to these causes in Brazil between 2002 and 2011: 2.1% annual increase in transportation accidents, 2.7% in falls, and 8.3% in other external causes^8^ . An increase in hospitalization due to falls is a result of the continuous increase in the number of older adults in Brazil and higher incidence in this population^8^ . A study on this theme identified an increasing trend in hospitalization and mortality rates in Brazil from 1996 to 2012. Hospital admissions increased from 2.58 to 41.37 per 10,000 older adults^17^ .

A study conducted by the Institute for Applied Economic Research (IPEA) showed 50.3% increase in traffic accidents on federal highways, 34.5% increase in deaths, and 50% increase in the number of victims^18^ . These data are similar to those found in this study, and although obtained through different methods, they are consistent with growing urbanization, increased number of vehicles, and consequently, traffic accidents in Brazil.

Other external causes that increased in the study period include the care complications and other accidents. Care complications refer to adverse events during care provision, and other accidents refer to occurrences at home, work, while practicing sports and leisure activities, inhalation, accidental poisoning and burns. The real dimension of these problems may be underestimated, since few Brazilian studies have addressed these issues. Considering the magnitude and growth of hospitalization rates due to trauma in Brazil, information from the SIH-SUS is essential because of its relevance as a cause of ICU morbidity.

Male patients, young and older adults were the groups with the highest hospitalization rates. A prevalence of male and young adults was obtained in Canada, where the ratio between sexes was 1.75 male admissions for each female admission^19^ . In this study, although an increased rate among female patients decreased the ratio between sexes, it does not represent greater male access to health services, but greater exposure to external causes, and then a greater risk of traffic and work accident^20 , 21^ . An increased rate among older adults can be explained by more frequent falls^17^ . The factors that led to an increase in ICU hospitalization due to trauma may include diversity of the structure of urgent and emergency services, a fact that may determine different access to hospital beds in each region and state of Brazil, and sociodemographic changes, urbanization and increase in the number of vehicles on public roads, which affect the quantity and severity of external causes.

Prompt and effective pre-hospital care (PHC) to diagnose and determine the complexity of a definitive treatment^22^ becomes crucial for the trauma victim to reach the ICU. In this context, the number of PHC sites was expanded in the urgent and emergency network. The emergency care units (UPA) increased from 101 in 2011 to 446 in 2016, proportionally higher in the Southeast region and lower in the North region^23^ . The Mobile Emergency Care Service (SAMU) expanded its coverage from 35% in 2003 to 75.9% in 2015, higher in the South region and lower in the North region^24^ . An articulated organization of urgent services can make the trauma service less fragmented by using, in addition to the PHC structures, secondary level units to stabilize victims and regulate care, and qualify urgent hospital care, increase the number of ICU beds and home care^25^ .

Just like the greater access to PHC, the expansion of ICU beds in Brazil influences ICU hospitalization due to trauma. In 2012, the proportion was 13 ICU beds for every 100,000 inhabitants^26^ , which increased to 21.9 in 2016^27^ , but with significant regional variation, for instance, 27.8 for every 100,000 inhabitants in the Southeast region and 13.7 for every 100,000 inhabitants in the North region. Significant regional differences have also been reported among countries. In the United States, the proportion is 25 ICU beds for every 100,000 inhabitants, while in Europe, 11.5 for every 100,000 inhabitants^28^ .

The difference in ICU hospitalization rates due to trauma between the regions shows a change in the supply of ICU beds in Brazil, which is suggested by the growing trend in the North and Northeast regions. The use of beds is closely related to bed supply and, in moments of high demand or limited resources, trauma patients may not be admitted. In addition, an increased number of beds in more populated regions may not be a response to population changes, but to financial pressure^29^ .

In Brazil, hospital services are planned according to scientific evidence, protocols, structure and profit analysis, consolidated examples and simulation models^30^ , but such planning does not consider the dynamics of access to beds and management specificities of each ICU^22^ . An adequate dimensioning of beds considers the number of expected admissions, reflecting an ideal proportion of beds for each age group, bed type and ICU^30^ . To ensure stability in the bed regulation system, waiting times and patient flow management should be priorities not only for access to ICU beds, but also for quality management of bed use^22^ .

In addition to the difference in the structure of PHC services and ICU beds among Brazilian regions, the difference in the magnitude of hospitalization rates due to trauma in these units can be attributed to greater urbanization, economic development and longevity in the South and Southeast regions^8^ . The possibility of unequal access and quality of health services should be considered, given the incidence of trauma in the population, the use of ICU beds and the definition of ICU bed in each region^19^ .

Also, differences between hospitals, hospitalization profiles and type of care provided^26^ may be related to the access and outcome of serious diseases in Brazil. In this sense, reduced mortality due to trauma in the ICU is a positive aspect, especially for those who survive before their contact with the APH and the hospital. SAMU implementation and structuring, specialization of urgent and emergency services, and improvement of equipment and procedures are determinants for changes in the profile of general hospital and ICU admission. Certainly, the quality of hospital care may have an impact on mortality, especially in late mortality, since interventions may have complex and variable effects during the hospitalization time^31^ .

Other factors may influence the hospital morbidity profile in ICU due to trauma and determine the impact on rates during the study years, such as aspects of society in the period, increase in income and life expectancy, improvements in social indicators and implementation of prevention policies for factors that are common to all types of trauma, such as traffic and mobility (Brazilian Traffic Code, City Statute and Urban Mobility Law), violence (National Policy for the Reduction of Morbidity and Mortality by Accidents and Violence) and care (National Patient Policy and Statute of Older Adults). Even with differences between Brazilian regions and states, these factors are relevant for social development and determine changes in individual and collective behaviors.

Between 1999 and 2013, Brazil presented a strong growth in the number of vehicles and motorcycles, which became a problem in the perspective of environmental sustainability, quality of life, and health, especially when public policies reinforce a pattern of mobility and urbanization linked with individual transport^32^ . This strong and fast urbanization without investments in infrastructure and industrialization generates social problems that are expressed in traffic accidents and violence, with collective and generational consequences.

This study emphasizes the importance of SUS databases, in this case SIH-SUS, as a tool to monitor the occurrence of trauma, determining an indirect and complementary interpretation of in-hospital care system and factors that influence the rates, such as social and demographic ones. Although some determinants influence ICU hospitalization due to trauma, further studies are required using different sources of data, exploring, for example, trauma severity of patients treated in emergency services or hospitals and sent to the ICU, as well as studies analyzing the external causes of such admissions.

The main study limitation refers to the heterogeneous quality of SIH-SUS data in the time series, since mistakes in filling demographic data and choosing a code for each external may influence its interpretation. The main conclusion of this study refers to an increase in hospital morbidity due to trauma in ICU in Brazil from 1998 to 2015, which contrasted with a reduction in general all-cause hospitalization. The increase trend was not homogeneous among Brazilian states and regions. The results demonstrated an increase in hospitalization due to trauma among female patients, although the prevalence is still among male patients, besides a significant increase in admissions among older adults.

The epidemiological context analyzed in this study shows the tragic situation of an increase in transport accidents and falls and the reality of ICU hospitalization due to trauma which is only the tip of the iceberg of trauma occurrence in Brazil. If this trend does not change, the demand for specialized beds will increase, with a high impact on society, due to consequences such as sequelae or death, and for the public sector, due to the need to expand ICU beds and facilities.
