# Supplementary material for: Epidemiological overview – 18 years of ICU hospitalization due to trauma in Brazil
Source: Rev Saude Publica. 2019 Sep 18;53:83. doi: 10.11606/s1518-8787.2019053001178 (PMC6763286; doi:10.11606/s1518-8787.2019053001178)
Supplement: Translation

# Translation: Panorama epidemiológico de dezoito anos de internações por trauma em UTI no Brasil

**Authors:** Maicon Henrique Lentsck, Ana Paula Sayuri Sato, Thais Aidar de Freitas Mathias

**Affiliations:** I Universidade Estadual de Maringá, Programa de Pós-Graduação em Enfermagem. Maringá, PR, Brasil.; II Universidade de São Paulo. Faculdade de Saúde Pública. Departamento de Epidemiologia. São Paulo, SP, Brasil.; III Universidade Estadual de Maringá. Departamento de Enfermagem. Maringá, PR, Brasil.

**Keywords:** Ferimentos e Lesões, epidemiologia, Unidades de Terapia Intensiva, tendências, Cuidados Críticos, Estudos de Séries Temporais

## Abstract

**OBJETIVO:**

Analisar a magnitude e a tendência das taxas de internação por lesões traumáticas em unidades de terapia intensiva (UTI) no Brasil de 1998 a 2015.

**MÉTODOS:**

Estudo ecológico de séries temporais com dados do Sistema de Informações Hospitalares. A análise de tendência das taxas de internação segundo diagnóstico, sexo e idade foi realizada por modelos de regressão linear generalizada e procedimento de Prais-Winsten.

**RESULTADOS:**

As taxas foram mais elevadas para os homens, mas o crescimento das internações por trauma para as mulheres influenciou a razão das taxas entre os sexos. As quedas e os acidentes de transporte foram as causas mais frequentes dos traumas. Houve aumento de 3,6% ao ano, em média, nas taxas de internação por trauma em UTI no Brasil, maior na região Norte (8%; IC95% 6,4–9,6), entre as mulheres (5,4%; IC95% 4,5–6,3) e pessoas com 60 anos ou mais (5,5%; IC95% 4,7–6,3). Entre as causas do trauma, as quedas (4,5%; IC95% 3,5–5,5) e complicações assistenciais (5,4%; IC95% 4,5–6,3) foram as mais importantes. Por outro lado, as taxas de mortalidade hospitalar por trauma em UTI declinaram 1,7% ao ano, em média (IC95% 2,1–1,3).

**CONCLUSÃO:**

O aumento das internações por trauma em UTI no Brasil pode ser resultado de alguns fatores, como a ocorrência crescente de acidentes e violências, a implementação do atendimento pré-hospitalar e também a melhoria no acesso à assistência, com maior número de leitos em UTI. Soma-se a isso o envelhecimento da população, pois observou-se aumento mais acentuado das internações em pessoas com 60 anos de idade ou mais.

## INTRODUÇÃO

Os traumas decorrentes de causas externas continuam a ser o principal motivo de morte e incapacidades em pessoas de 5 a 29 anos^1^ , embora o envelhecimento possa estar influenciando o aumento das taxas de internação por trauma em idosos. Na Espanha, entre 2000 e 2010, o aumento anual nas taxas em idosos foi de 1,1% para homens e 0,9% para mulheres^2^ ; nos EUA, as internações por trauma representaram 4,4% do total entre 2000 e 2011, com declínio para crianças e jovens e estabilidade para idosos^3^ . Em 2015, o trauma representou 10,1% da carga global de doenças^4^ . A depender da gravidade, ele ocasiona sequelas ou até a morte.

Em 2017, o estudo da carga global de doenças, lesões e fatores de risco ( *Global Burden of Disease* – GBD) estimou que somente traumas não fatais por quedas e acidentes de trânsito geraram incapacidades em curto e longo prazo em 226,2 milhões de pessoas^5^ . Já as lesões fatais representaram 8% da mortalidade no mundo, vitimando 4,48 milhões de pessoas, com um aumento de 2,3% em relação aos dados de 2007 e uma taxa de mortalidade global de 57,9 por 100.000 habitantes^6^ . Nesse mesmo ano, o trauma foi responsável por 11,9% dos 1,65 bilhões de anos potenciais de vida perdidos^6^ .

Além do incremento, existe tendência de elevação na gravidade das lesões traumáticas. No Canadá, de 2002 a 2009 o crescimento das internações por traumas graves em pessoas com 65 anos de idade ou mais foi de 22% e de 10% entre os jovens^7^ . A tendência de intensificação da gravidade nos diferentes tipos de trauma também ocorreu na Espanha^2^ . O acréscimo de traumas graves exige aprimoramento na organização dos serviços de saúde e especialização da equipe multiprofissional, sendo necessária internação em unidades de terapia intensiva (UTI). Para conhecer a morbidade por trauma no Brasil, analisou-se o Sistema de Informações Hospitalares (SIH) do Sistema Único de Saúde (SUS), o banco de dados sobre internações no país, e observou-se que as hospitalizações por causas externas aumentaram 37,3% entre 2002 e 2011, com predomínio das quedas (41%) e dos acidentes de trânsito (15%)^8^ .

Parte importante das vítimas de trauma precisa de cuidado intensivo, essencial para sua sobrevivência^9^ . Nos Estados Unidos, em 2013, 33,8% dos traumatizados foram internados em UTI, com incidência maior em pessoas com 80 anos de idade ou mais (7,8 para cada 1.000 habitantes)^10^ . Estudar essas hospitalizações em UTI torna-se imprescindível para o planejamento e organização da assistência ao trauma e para o desenvolvimento de programas de prevenção^11^ .

Com o aumento dos acidentes de trânsito e da violência, principalmente das agressões^12^ , em especial nos grandes aglomerados urbanos, é possível que as internações por traumas em UTI apresentem também tendência crescente. O cuidado intensivo, especialmente as internações, representam proporcionalmente o maior custo da rede de atenção para o setor saúde e para a sociedade. Considerando o fardo das lesões traumáticas no perfil de morbimortalidade na população e que parte importante das vítimas é hospitalizada, entende-se que as taxas de internação em UTI devem ser analisadas no Brasil, identificando-se as tendências desse evento em regiões e estados, assim como o perfil por sexo, idade, tipo de causa externa que originou a internação, tempo de permanência e mortalidade hospitalar. Esses estudos podem servir de apoio para o planejamento e monitoramento de políticas públicas. Portanto, o objetivo deste estudo foi analisar a magnitude e a tendência das taxas de internação em UTI por lesões traumáticas no Brasil de 1998 a 2015.

## MÉTODOS

Estudo ecológico de séries temporais com dados de internações hospitalares por trauma em UTI financiadas pelo SUS, para as competências de apresentação das autorizações de internação hospitalar (AIH) no SIH-SUS de janeiro de 1998 a dezembro de 2015 no Brasil. A escolha do início da série se justifica pela utilização da 10ª revisão da Classificação Internacional de Doenças (CID-10) para morbidade hospitalar no Brasil e pela época da publicação da portaria que instituiu a exigência de registro do código da natureza da lesão no campo “diagnóstico principal” e da causa externa no “diagnóstico secundário” na AIH^13^ . O final da série retrata as últimas internações disponibilizadas no momento da coleta de dados.

Por meio do endereço eletrônico do Departamento de Informática do SUS (DATASUS), foram gerados arquivos mensais para cada ano da série e estado. Foram selecionados os registros com menção de uso de UTI e com diagnóstico principal de lesões e envenenamentos (capítulo XIX da CID-10) nos agrupamentos: traumas da cabeça (S00–S09); pescoço (S10–S19); tórax (S20–S29); abdome, dorso, coluna lombar e pelve (S30–S39); membros superiores (S40–S69); membros inferiores (S70–S99); múltiplas regiões do corpo (T00–T07); regiões não especificadas (T08–T14); efeito de corpo estranho através de orifício natural (T15–T19); queimaduras e corrosões (T20–T32); efeitos do frio (T33–T35); intoxicação por drogas, medicamentos e substâncias biológicas (T36–T50); efeitos tóxicos de substâncias não medicinais (T51–T65); outros efeitos de causas externas (T66–T78); algumas complicações precoces de traumatismo (T79); complicações de cuidados médicos e cirúrgicos (T80–T88) e sequelas de traumatismos (T90–T98). Esta seleção resultou em um banco de dados com 790.884 internações. Os dados de população foram coletados no endereço eletrônico do Instituto Brasileiro de Geografia e Estatística (IBGE), com base nos censos populacionais, contagens populacionais e estimativas intercensitárias.

As internações por lesões traumáticas foram analisadas por meio de números absolutos, relativos e diferença relativa. Foram calculadas taxas por 100.000 habitantes, estratificadas por sexo, idade e causa externa, segundo regiões e estados, padronizadas pelo método direto, tendo como referência a população do censo de 2010. As internações por causas externas correspondem ao diagnóstico secundário e são codificadas no capítulo XX da CID-10 (V01–Y98). Houve perda de 7.542 dessas internações (0,9%) por falta de registro. Calcularam-se as médias de permanência e taxas de mortalidade hospitalar a cada 100 internações por lesões traumáticas.

A análise da tendência foi realizada por meio de regressão linear generalizada, que considera as taxas de internação como variável dependente (Y) e os anos-calendário como variável independente (X). A fim de corrigir o efeito da autocorrelação temporal de primeira ordem dos resíduos, utilizou-se o procedimento de Prais-Winsten, o qual, pelo teste de Durbin-Watson, avalia a manifestação da autocorrelação. A interpretação do teste se dá por uma medida que varia de 0 a 4, em que 2 significa inexistência de autocorrelação serial^14^ .

As séries foram suavizadas por média móvel de terceira ordem. Posteriormente, foi realizada sua transformação logarítmica e construídos diagramas de dispersão e autocorrelação. Foi aplicado o modelo autorregressivo de Prais-Winsten, que identificou se o comportamento das taxas foi estável (p > 0,05), decrescente (p < 0,05 e coeficiente de regressão β_1_negativo) ou crescente (p < 0,05 e coeficiente de regressão β_1_positivo). Após a modelagem foi calculada a variação média percentual anual das taxas de internação no período para o coeficiente de regressão por meio do cálculo (-1 + 10^^^b)x100, e os respectivos intervalos de confiança de 95% (IC95%), pela fórmula b±tEP, onde t é o valor tabelado do teste t e EP o valor do erro-padrão do coeficiente de regressão^15^ . As tabelas foram apresentadas com os dados de 1998, 2007 e 2015, e a diferença relativa calculada entre os anos extremos. Para a tendência, foi utilizado o Stata (versão 13). O estudo foi dispensado de análise pelo comitê de ética em pesquisa, conforme Portaria nº 466 de 2012 do Conselho Nacional de Saúde, por se tratarem de dados secundários e de acesso público.

## RESULTADOS

Entre 1998 e 2015, enquanto as taxas de internação por todas as causas no Brasil pelo SUS diminuíram 28,5%, as internações em UTI aumentaram 21,4% ( Tabela 1 ). Quando as taxas de internação em UTI são analisadas por diagnóstico principal, observa-se crescimento maior para afecções originadas no período perinatal (acréscimo de 173,8%), seguidas das internações por traumas, com 91,8% de aumento ( Tabela 2 ). As internações por traumas aumentaram principalmente nas regiões Norte e Nordeste – 296,9% e 167,1%, respectivamente. Já a mortalidade hospitalar diminuiu 25,1%, destacando-se a região Norte, com queda de 33,5% ( Tabela 1 ). Os traumas de cabeça, quadril e coxa foram os mais frequentes, com taxas de 9,7 e 6,9 por 100.000 habitantes, respectivamente. Os maiores aumentos no período estudado foram das queimaduras e corrosões (4.566,7%) e complicações de cuidados médicos e cirúrgicos (466,7%) ( Tabela 2 ).

**Tabela 1:** Taxas de internação hospitalar por todas as causas e clínicas, por todas as causas em UTI e por trauma em UTI e taxas de mortalidade por trauma em UTI, segundo grandes regiões e ano. Brasil, 1998, 2007 e 2015.

Internações	1998		2007		2015		Dif. rel.^b^
		
	n	Taxa^a^	n	Taxa^a^	n	Taxa^a^	
Todas as causas, todas as clínicas							

Brasil	12.248.631	7.717,2	11.733.572	6.052,6	11.623.829	5.515,1	-28,5
Norte	851.777	7.176,6	996.349	6.494,0	951.726	5.447,0	-24,1
Nordeste	3.601.756	7.962,2	3.277.182	6.278,9	3.120.990	5.518,0	-30,7
Sudeste	4.904.176	7.111,5	4.671.206	5.792,6	4.660.086	5.434,8	-23,6
Sul	2.020.358	8.364,4	1.873.754	6.778,8	2.003.708	6.854,9	-18,0
Centro-Oeste	870.564	7.917,9	915.081	6.770,3	887.319	5.746,1	-27,4

Todas as causas em UTI							

Brasil	381.642	275,7	511.989	278,1	705.181	334,8	21,4
Norte	5.911	73,5	19.796	158,4	29.996	215,4	193,1
Nordeste	65.740	165,4	91.134	178,3	138.643	248,7	50,4
Sudeste	178.310	293,7	246.763	302,7	334.322	336,2	14,5
Sul	98.640	456,8	119.607	431,0	155.431	484,9	6,2
Centro-Oeste	33.041	392,8	34.689	286,2	46.789	317,4	-19,2

Trauma^c^em UTI							

Brasil	24.883	17,1	42.834	23,4	67.705	32,8	91,8
Norte	606	6,5	1.427	10,9	3.810	25,8	296,9
Nordeste	3.003	7,3	6.280	12,7	10.763	19,5	167,1
Sudeste	13.175	21,0	23.029	28,6	33.268	36,0	71,4
Sul	5.905	26,4	9.725	35,4	15.567	50,4	90,9
Centro-Oeste	2.144	22,8	2.373	18,7	4.297	29,0	27,2

Mortalidade hospitalar	n	Taxa^d^	n	Taxa^d^	n	Taxa^d^	Dif. rel.^b^

Trauma em UTI							

Brasil	5.349	21,5	8.626	20,1	10.920	16,1	-25,1
Norte	170	28,1	294	20,6	711	18,7	-33,5
Nordeste	781	26,0	1.598	25,4	2.011	18,7	-28,1
Sudeste	2.904	22,0	4.407	19,1	5.079	15,3	-30,5
Sul	1.117	18,9	1.750	18,0	2.302	14,8	-21,7
Centro-Oeste	377	17,6	577	24,3	817	19,0	8,0

^a^Taxas padronizadas pela população brasileira do censo populacional de 2010, por 100.000 habitantes/ano.

^b^Diferença relativa entre as taxas dos anos extremos, 1998 e 2015.

^c^Diagnóstico principal de internação no capítulo XIX (lesões, envenenamentos e algumas outras consequências de causas externas, códigos S00–T98) da 10ª revisão da Classificação Internacional de Doenças (CID-10)

^d^Taxa de mortalidade hospitalar: razão entre o número de óbitos por trauma em UTI e as internações por trauma em UTI, multiplicada por 100.

**Tabela 2:** Taxas de internação por todas as causas em UTI segundo diagnóstico principal de internação e ano. Brasil, 1998, 2007 e 2015.

Diagnóstico principal^a^	1998		2007		2015		Dif. rel.^c^
		
	n	Taxa^b^	n	Taxa^b^	n	Taxa^b^	
Doenças do aparelho circulatório	129.248	105,7	156.919	90,8	193.810	89,0	-15,8
Doenças do aparelho respiratório	51.917	37,0	67.013	36,2	83.986	39,6	7,0
Afecções perinatais	31.269	14,1	66.631	29,8	75.492	38,6	173,8
Lesões e envenenamentos	24.833	17,1	42.834	23,4	67.705	32,8	91,8
Cabeça	10.822	7,1	16.497	8,9	20.145	9,7	36,6
Quadril e coxa	3.204	2,8	6.613	3,8	14.725	6,9	146,4
Abdome	2.057	1,4	3.995	2,1	5.468	2,7	92,9
Múltiplas regiões do corpo	2.244	1,5	646	0,3	1.904	0,9	-40,0
Intoxicações não medicinais	1.285	0,8	1.535	0,8	1.370	0,7	-12,5
Intoxicações por drogas	1.165	0,7	1.599	0,9	1.248	0,6	-14,3
Complicações assistenciais^d^	830	0,6	3.275	1,8	7.267	3,4	466,7
Tórax	910	0,6	1.825	1,0	4.582	2,2	266,7
Queimaduras e corrosões	73	0,03	1.950	1,0	2.925	1,4	4.566,7
Pescoço	441	0,3	1.144	0,6	1.393	0,7	133,2
Demais agrupamentos^e^	1.802	1,2	3.755	2,1	6.678	3,2	166,7
Neoplasias	19.768	15,2	40.741	23,0	59.893	27,8	82,9
Demais capítulos da CID-10	124.607	86,6	137.851	74,8	224.295	106,5	23,0

Total	381.642	275,7	511.989	278,1	705.181	334,8	21,4

CID-10: 10ª revisão da Classificação Internacional de Doenças

^a^Por capítulos da CID-10 e agrupamentos do capítulo XIX, sobre lesões e envenenamentos.

^b^Taxas padronizadas pela população brasileira do censo populacional de 2010, por 100.000 habitantes/ano.

^c^Diferença relativa entre as taxas dos anos extremos, 1998 e 2015.

^d^Complicações de cuidados médicos e cirúrgicos não classificados em outra parte.

^e^Traumas de ombro e braço; cotovelo e ombro; punho e mão; joelho e perna; tornozelo e pé; localização não especificada; por penetração de corpo estranho em orifícios naturais; queimaduras e corrosões; geladuras; outros efeitos de causas não especificadas; algumas complicações precoces de traumas e sequelas de traumas, intoxicações e outras consequências.

As taxas de internação por trauma em UTI no Brasil quase dobraram, passando de 17,1/100.000 habitantes em 1998 para 32,8/100.000 habitantes em 2015, um aumento médio de 3,6% ao ano (IC95% 2,8–4,3) ( Figura ). Mesmo apresentando menores taxas de internação, o crescimento foi maior para a região Norte (8,0% ao ano, IC95% 6,4–9,6) e para os estados de Roraima (21,7% ao ano, IC95% 7,4–37,8), Bahia (12,7% ao ano, IC95% 9,3–16,3) e Rondônia (11,9% ao ano, IC95% 0,0–25,3). Apenas para o estado da Paraíba houve declínio de 2,8% ao ano (IC95% 4,2–1,3). Não houve tendência das taxas para a região Centro-Oeste (IC95% -1,3–3,3), devido à oscilação das altas taxas no estado de Goiás. As regiões Sul e Sudeste detêm as mais elevadas taxas de internação no país, com 50,4/100.000 e 36,0/100.000 habitantes, respectivamente, em 2015. Mesmo com aumento médio anual 3,3%, menor do que os observados para as regiões Norte e Nordeste (8% e 5,3% respectivamente), chamam atenção, nas regiões Sul e Sudeste, as taxas elevadas no Paraná, Santa Catarina, São Paulo e Minas Gerais (69,1; 45,6; 42,3 e 39,8, respectivamente) em 2015 ( Tabela 3 , Figura ).

**Figura:** Taxas de internação por 100.000 habitantes por trauma em UTI. Brasil e regiões, 1998–2015.

**Tabela 3:** Taxas de internação por trauma em UTI, variação média anual, intervalo de confiança (IC95%) e tendência. Brasil, grandes regiões e estados, 1998 a 2015.

Brasil, região e estado	Taxa^a^			Variação anual^b^	IC95%	Tendência

	1998	2007	2015			
Brasil	17,1	23,4	32,8	3,6	2,8 – 4,3	Crescente
Norte	6,5	10,9	25,8	8,0	6,4 – 9,6	Crescente
Acre	8,5	22,5	37,7	3,6	1,7 – 5,6	Crescente
Amapá	6,5	11,8	13,2	6,2	-2,9 – 16,2	-
Amazonas	2,0	8,9	12,5	11,4	5,0 – 18,1	Crescente
Pará	5,3	8,8	25,5	11,0	8,6 – 13,3	Crescente
Rondônia	6,5	2,4	37,9	11,9	0,0 – 25,3	Crescente
Roraima	0,0	20,6	33,4	21,7	7,4 – 37,8	Crescente
Tocantins	14,3	15,9	34,4	5,4	0,2 – 10,8	Crescente
Nordeste	7,3	12,7	19,5	5,3	3,7 – 6,8	Crescente
Alagoas	7,4	16,7	17,0	1,7	-2,0 – 5,4	-
Bahia	2,1	10,6	21,0	12,7	9,3 – 16,3	Crescente
Ceará	12,7	16,5	14,4	0,5	-2,7 – 3,8	-
Maranhão	3,0	7,6	17,3	9,5	4,6 – 14,7	Crescente
Paraíba	31,4	28,4	22,0	-2,8	-4,2 – -1,3	Decrescente
Pernambuco	4,0	5,9	25,2	10,4	3,9 – 17,2	Crescente
Piauí	5,7	9,4	18,0	7,0	3,5 – 10,6	Crescente
Rio Grande do Norte	4,2	28,3	14,1	7,9	0,6 – 15,8	Crescente
Sergipe	9,0	10,4	22,5	5,4	-0,0 – 11,1	-
Sudeste	21,0	28,6	36,0	3,3	2,8 – 3,8	Crescente
Espírito Santo	13,3	22,9	33,0	5,3	2,9 – 7,9	Crescente
Minas Gerais	19,2	29,0	42,3	4,9	3,7 – 6,0	Crescente
Rio de Janeiro	12,9	9,6	19,1	2,2	-1,4 – 6,0	-
São Paulo	25,8	36,5	39,8	2,5	1,7 – 3,2	Crescente
Sul	26,4	35,4	50,4	3,3	2,5 – 4,1	Crescente
Paraná	39,4	44,5	69,1	3,5	1,8 – 5,3	Crescente
Rio Grande do Sul	16,8	26,6	38,4	3,0	1,3 – 4,7	Crescente
Santa Catarina	23,2	36,8	45,6	3,6	2,4 – 4,8	Crescente
Centro-Oeste	22,8	18,7	29,0	1,0	-1,3 – 3,3	-
Distrito Federal	7,6	11,6	16,4	5,3	2,9 – 7,9	Crescente
Goiás	36,2	21,0	34,0	-1,2	-4,5 – 2,2	-
Mato Grosso	9,1	18,5	31,0	6,4	4,7 – 8,2	Crescente
Mato Grosso do Sul	18,9	20,5	28,2	2,6	0,5 – 4,8	Crescente

^a^Taxas padronizadas pela população brasileira do censo populacional de 2010, por 100.000 habitantes/ano.

^b^Variação percentual média anual das taxas de internação calculada a partir do β_1_do modelo de regressão linear generalizada de Prais-Winsten^11^.

A taxas de internação por traumas em UTI são duas vezes mais elevadas para os homens, mas o aumento foi maior para as mulheres (em média, 5,4% ao ano, IC95% 4,5–6,3) do que para eles (3,8% ao ano, IC95% 3,1–4,5), o que influenciou a diminuição da razão das taxas no período. O crescimento médio anual foi maior para pessoas de 60 anos de idade ou mais (5,5%, IC95% 4,7–6,3) ( Tabela 4 ).

**Tabela 4:** Taxas de internação por trauma em UTI, segundo sexo, idade, causa externa da lesão, média de permanência e mortalidade hospitalar, variação média anual, intervalo de confiança (IC95%) e tendência. Brasil, 1998–2015.

Características	Taxa^a^			Variação anual^b^	IC95%	Tendência

	1998	2007	2015			
Sexo						

Masculino	22,2	32,2	44,3	3,8	3,1 – 4,5	Crescente
Feminino	8,7	13,4	22,2	5,4	4,5 – 6,3	Crescente
Razão M/F	2,6	2,3	2,0	-1,5	-2,1 – -1,0	Decrescente

Idade (anos)						

Até 14	6,4	9,0	10,6	2,5	1,7 – 3,3	Crescente
15–29	17,4	23,3	29,7	3,2	2,5 – 3,8	Crescente
30–59	16,6	22,5	28,3	2,8	2,1 – 3,5	Crescente
60 ou mais	39,3	58,8	101,4	5,5	4,7 – 6,3	Crescente

Causa externa da lesão^c^						

Complicações assistenciais (Y40–Y84)	0,9	1,4	1,9	5,1	3,9 – 6,4	Crescente
Outros traumas acidentais (W20–X59)	3,0	4,2	7,0	4,6	3,4 – 5,8	Crescente
Quedas (W00–W19)	5,0	7,6	10,8	4,5	3,5 – 5,5	Crescente
Sequelas de causas externas (Y85–Y89)	0,6	0,7	1,2	4,5	1,8 – 7,3	Crescente
Demais agrupamentos (Y35–Y36; Y90–Y98)	0,1	0,1	0,2	4,4	2,0 – 6,8	Crescente
Eventos de intenção indeterminada (Y10–Y34)	1,0	1,2	2,5	4,2	1,5 – 7,0	Crescente
Lesões autoprovocadas intencionais (X60–X84)	0,2	0,5	0,5	4,0	1,5 – 6,7	Crescente
Acidentes de transporte (V01–V99)	3,4	5,4	6,8	3,7	2,9 – 4,4	Crescente
Pedestre (V01–V09)	0,8	1,5	1,3	4,0	2,3 – 5,9	Crescente
Ciclista (V10–V19)	2,0	3,1	3,5	3,2	2,8 – 3,6	Crescente
Motociclista (V20–V29)	1,2	1,8	2,8	4,3	3,1 – 5,5	Crescente
Ocupante de veículo (V30–V79)	0,6	0,8	1,0	3,3	1,5 – 5,1	Crescente
Outros (V80–V99)	0,6	1,0	1,4	3,1	0,2 – 6,1	Crescente
Agressões (X85–Y09)	1,1	1,4	2,2	3,2	1,5 – 4,9	Crescente

Média em dias de permanência em UTI	4,9	6,1	6,3	1,5	1,0 – 2,1	Crescente
Taxa de mortalidade hospitalar em UTI	21,5	20,1	16,1	-1,7	-2,1 – -1,3	Decrescente

Razão M/F: razão entre os sexos masculino e feminino

^a^Taxas padronizadas pela população brasileira do censo populacional de 2010, por 100.000 habitantes/ano.

^b^Variação percentual média anual das taxas de internação calculadas a partir do β_1_do modelo de regressão linear generalizada de Prais-Winsten^11^.

^c^Diagnóstico secundário de internação, capítulo XX da 10ª revisão da Classificação Internacional de Doenças (CID-10).

As quedas, acidentes de transporte e outros traumas acidentais foram as causas externas que mais provocaram internações em UTI no ano de 2015, com taxas de 10,8; 6,8 e 7,0/100.000 habitantes, respectivamente. As taxas de internação de motociclistas e pedestres foram as que mais aumentaram (4,3% e 4%, respectivamente). Todas as causas externas que motivaram internação em UTI apresentaram crescimento no período, como as complicações assistenciais (5,1%), outros traumas acidentais (4,6%) e as quedas (4,5%). Houve aumento na média de permanência em UTI (1,5% ao ano, IC95% 1,0–2,1); entretanto, a mortalidade hospitalar diminuiu em média 1,7% ao ano (IC95% 2,1–1,3) ( Tabela 4 ).

## DISCUSSÃO

A análise epidemiológica das hospitalizações financiadas pelo SUS no Brasil mostrou que houve declínio das internações por todas as causas no período, mas houve aumento daquelas em UTI. De 1998 a 2015, o trauma representou a quarta causa de internações em UTI. Com exceção das afecções perinatais, o crescimento relativo das taxas de internação por trauma nessas unidades foi o mais elevado e ocorreu em todas as regiões do país, com médias anuais de crescimento maiores para a Norte e menores para a Centro-Oeste. Outra modificação foi observada em relação ao sexo e à idade, pois o aumento médio das taxas foi maior para mulheres e idosos.

Apesar das características heterogêneas do perfil de morbimortalidade populacional, de variação da estrutura e capacidade das UTI e da organização do atendimento em saúde^10^ , as internações por trauma em UTI no sistema público de saúde brasileiro assemelham-se a dados internacionais^16^ . Estudo suíço sobre hospitalizações intensivas, entre 2008 e 2012, identificou que os traumas representaram 44,3% do total, sendo mais prevalentes os cerebrais (64,4%) e torácicos (59,8%), sendo os acidentes de trânsito (40,4%) e as quedas (34,4%) as principais causas^16^ .

O aumento observado nas causas externas de internação por trauma em UTI observado é semelhante ao perfil de morbidade hospitalar por essas causas no Brasil entre 2002 e 2011: aumento anual de 2,1% para acidentes de transporte, 2,7% para quedas e 8,3% para demais causas externas^8^ . O aumento das internações por quedas destaca-se pelo contínuo aumento de idosos na população brasileira e da incidência do agravo nessa população^8^ . Estudo sobre o tema identificou tendência crescente nas taxas de internação e de mortalidade no Brasil de 1996 a 2012. As internações aumentaram de 2,58 para 41,37 a cada 10.000 idosos^17^ .

Levantamento do Instituto de Pesquisa Econômica Aplicada (Ipea) mostrou aumento de 50,3% nos acidentes de trânsito em rodovias federais, acréscimo de 34,5% nas mortes e de 50% nos feridos^18^ . Dados similares aos identificados nesse estudo, embora com métodos diferentes, apresentam-se coerentes com a crescente urbanização, aumento no número de veículos e consequente aumento nos acidentes de trânsito no Brasil.

Outras causas externas que aumentaram foram as complicações assistenciais e outras causas acidentais. As primeiras abrangem eventos adversos ocorridos durante a prestação de serviço de saúde, e as segundas, outros eventos acidentais, como os domésticos, de trabalho, de práticas esportivas e lazer, as inalações, os envenenamentos acidentais e as queimaduras. A real dimensão desses problemas pode estar subestimada, pois são poucos os estudos brasileiros que os abordam. Diante da magnitude e crescimento das taxas de internação por trauma no Brasil, informações advindas do SIH-SUS tornam-se fundamentais por sua relevância enquanto causa de morbidade em UTI.

Indivíduos do sexo masculino, adultos jovens e idosos, compuseram os grupos com maiores taxas de internação. A predominância masculina e de adultos jovens para essas internações foi demonstrada no Canadá, em que a razão entre os sexos foi de 1,75 internação masculina para cada uma feminina^19^ . Embora no presente estudo o crescimento das taxas entre as mulheres tenha diminuído a razão entre os sexos, isso não representa maior acesso masculino aos serviços de saúde, mas maior exposição às causas externas, com maior propensão dos homens a acidentes de trânsito e de trabalho^20 , 21^ . O aumento das taxas entre idosos, por sua vez, pode ser explicado pela maior ocorrência de quedas^17^ . Os fatores que levaram ao aumento das internações por trauma em UTI podem compreender desde a diversidade na estrutura dos serviços de urgência e emergência – fato que pode determinar acesso diverso aos leitos hospitalares em cada região e estado brasileiro – às mudanças sociodemográficas, urbanização e crescimento da frota de veículos nas vias públicas, que, por sua vez, interferem na quantidade e gravidade das causas externas.

A exigência de atendimento pré-hospitalar (APH) rápido e eficaz para estabilizar, diagnosticar e determinar a complexidade de tratamento definitivo^22^ torna-se determinante para que a vítima de trauma chegue até a UTI. Nesse contexto, houve expansão dos pontos de atenção do APH na rede de urgência e emergência. As unidades de pronto atendimento (UPA) cresceram de 101 em 2011 para 446 em 2016, proporcionalmente em maior quantidade na região Sudeste e menor na Norte^23^ . O Serviço de Atendimento Móvel de Urgência (SAMU) passou de uma cobertura de 35% em 2003 para 75,9% em 2015, sendo maior na região Sul e menor na Norte^24^ . A organização dos serviços de urgência de forma articulada em rede pode tornar o atendimento ao traumatizado menos fragmentado ao utilizar, além das estruturas de APH, unidades de nível secundário para estabilizar vítimas e regular o atendimento, além da qualificação do atendimento de urgência hospitalar, ampliação de leitos de UTI e atenção domiciliar^25^ .

Assim como o aumento do acesso ao APH, a expansão de leitos intensivos no Brasil influencia as internações por trauma em UTI. Em 2012, havia 13 leitos de UTI por 100.000 habitantes^26^ , índice que passou para 21,9/100.000 em 2016^27^ , porém com desigualdades regionais importantes: 27,8/100.000 na região Sudeste e 13,7/100.000 na Norte. Mudanças regionais também são identificadas entre países com variações consideráveis. Nos EUA existem 25 leitos de UTI por 100.000 habitantes, enquanto na Europa são 11,5/100.000^28^ .

Considera-se que a diferença das taxas de internação por trauma em UTI entre as regiões demonstra uma mudança na oferta de leitos de UTI no Brasil, o que é sugerido pela tendência crescente nas regiões Norte e Nordeste. A utilização dos leitos muitas vezes é resultado da oferta e, em momentos da alta demanda ou limitação de recursos, traumatizados podem não ser admitidos. Além disso, o aumento de leitos em regiões mais populosas por vezes não é uma resposta a mudanças populacionais, mas sim a pressões financeiras^29^ .

No Brasil, os parâmetros de planejamento dos serviços hospitalares seguem evidências científicas, protocolos, análises de estrutura e rendimentos, exemplos consolidados e modelos de simulação^30^ , porém não consideram a dinâmica de acesso aos leitos e as especificidades de gestão em cada UTI^22^ . Para o dimensionamento adequado de leitos, leva-se em conta o número de internações esperadas, que se reflete em uma proporção ideal de leitos, dividida por faixas etárias, tipo de leito e UTI^30^ . Já para garantir a estabilidade no sistema de regulação de leitos, o tempo de espera e a gestão do fluxo de pacientes devem ser considerados prioridades não só para o acesso ao leito intensivo, mas também para gerenciar sua ocupação com qualidade^22^ .

Além da diferença na estrutura dos serviços de APH e de leitos de UTI entre as regiões brasileiras, a diferença na magnitude das taxas de internação por trauma nessas unidades pode ser atribuída à maior urbanização, desenvolvimento econômico e longevidade nas regiões Sul e Sudeste^8^ . A possibilidade de desigualdade de acesso e qualidade dos serviços de saúde pode ser aventada, dada a incidência do agravo nas populações, a forma de ocupação dos leitos de UTI e a própria definição de leito intensivo em cada região^19^ .

Considera-se ainda que as diferenças entre hospitais, perfis de internação e tipo da assistência prestada^265^podem estar relacionadas com o acesso e o desfecho das doenças graves no Brasil. Nesse sentido, é positivo o decréscimo na mortalidade por traumas em UTI, sobretudo para os que sobrevivem até o contato com o APH e o hospital. A implantação e estruturação do SAMU, especialização dos serviços de urgência e emergência e melhora nos equipamentos e procedimentos são fatores determinantes para a mudança no perfil de internação hospitalar em geral e em UTI. Certamente a qualidade do cuidado hospitalar oferecido também pode impactar na mortalidade, sobretudo na mortalidade tardia, pois as intervenções podem ter efeitos complexos e variáveis no tempo de internação^31^ .

Outros fatores podem influenciar o perfil de morbidade hospitalar em UTI por traumas e determinar impacto nas taxas ao longo dos anos estudados, como aspectos da sociedade no período, aumento da renda e expectativa de vida, melhorias de indicadores sociais e implementação de políticas de prevenção aos fatores comuns a todos os traumas, como sobre o trânsito e mobilidade (Código de Trânsito Brasileiro, Estatuto da Cidade e Lei de Mobilidade Urbana), violência (Política Nacional de Redução da Morbimortalidade por Acidentes e Violências) e cuidado (Política Nacional de Segurança do Paciente e Estatuto do Idoso). Mesmo com diferenças entre as regiões e estados brasileiros, esses fatores são relevantes para o desenvolvimento social e determinam mudanças no comportamento individual e coletivo.

Entre 1999 e 2013, o Brasil apresentou grande aumento no número de automóveis e motocicletas, o que se tornou um problema do ponto de vista da sustentabilidade ambiental, qualidade de vida e saúde, sobretudo quando políticas públicas reforçam um padrão de mobilidade e urbanização vinculado ao transporte individual^32^ . Essa urbanização forte e rápida sem investimentos na infraestrutura e industrialização gera problemas sociais que se expressam em acidentes de trânsito e violências, com consequências coletivas e geracionais.

Este estudo reitera a importância dos bancos de dados do SUS – neste caso, o SIH-SUS – como ferramenta de monitoramento das ocorrências do trauma, determinando interpretação indireta e complementar do sistema de atendimento intra-hospitalar e de fatores que interferem nas taxas, como os sociais e demográficos. Mesmo que alguns determinantes influenciem nas internações por trauma em UTI, são necessárias análises futuras sobre eles com diversas fontes de dados, explorando, por exemplo, a gravidade do trauma de indivíduos atendidos em prontos-socorros ou em hospitais de referência que são encaminhados para a UTI, assim como análises somente das causas externas dessas internações.

Destaca-se a qualidade heterogênea dos dados do SIH-SUS ao longo da série como a principal limitação do estudo, já que erros de preenchimento dos dados demográficos e da codificação das causas externas podem influenciar em sua interpretação. A principal conclusão deste estudo diz respeito ao aumento da morbidade hospitalar por trauma em UTI no Brasil no período de 1998 a 2015, que contrastou com a diminuição das internações hospitalares gerais por todas as causas. A tendência de aumento observada não foi homogênea entre estados e regiões brasileiras. Os resultados evidenciaram aumento de internações por trauma em mulheres, embora o predomínio seja ainda em homens, além de crescimento importante de internações em idosos.

O panorama epidemiológico analisado mostra a trágica situação do aumento dos acidentes de transporte e das quedas e remete à realidade de que as internações por traumas em UTI representam apenas a ponta do *iceberg* da ocorrência de traumas no Brasil. Se essa tendência não mudar, a demanda por leitos especializados será crescente, com alto impacto para a sociedade, devido às consequências como sequelas ou óbito, e para o setor público, pela necessidade de aumento das instalações e leitos de UTI.
